# Two-enzyme systems for glycolipid and polyglycerolphosphate lipoteichoic acid synthesis in *Listeria monocytogenes*

**DOI:** 10.1111/j.1365-2958.2009.06829.x

**Published:** 2009-08-24

**Authors:** Alexander J Webb, Maria Karatsa-Dodgson, Angelika Gründling

**Affiliations:** Department of Microbiology, Imperial College LondonSouth Kensington Campus, London SW7 2AZ, UK.

## Abstract

Lipoteichoic acid (LTA) is an important cell wall polymer in Gram-positive bacteria and often consists a polyglycerolphosphate backbone chain that is linked to the membrane by a glycolipid. In *Listeria monocytogenes* this glycolipid is Gal-Glc-DAG or Gal-Ptd-6Glc-DAG. Using a bioinformatics approach, we have identified *L. monocytogenes* genes predicted to be involved in glycolipid (*lmo2555* and *lmo2554*) and LTA backbone (*lmo0644* and *lmo0927*) synthesis. LTA and glycolipid analysis of wild-type and mutant strains confirmed the function of Lmo2555 and Lmo2554 as glycosyltransferases required for the formation of Glc-DAG and Gal-Glc-DAG. Deletion of a third gene, *lmo2553*, located in the same operon resulted in the production of LTA with an altered structure. *lmo0927* and *lmo0644* encode proteins with high similarity to the staphylococcal LTA synthase LtaS, which is responsible for polyglycerolphosphate backbone synthesis. We show that both proteins are involved in LTA synthesis. Our data support a model whereby Lmo0644 acts as an LTA primase LtaP and transfers the initial glycerolphosphate onto the glycolipid anchor, and Lmo0927 functions as LTA synthase LtaS, which extends the glycerolphosphate backbone chain. Inactivation of LtaS leads to severe growth and cell division defects, underscoring the pivotal role of LTA in this Gram-positive pathogen.

## Introduction

**Fig. 1:**
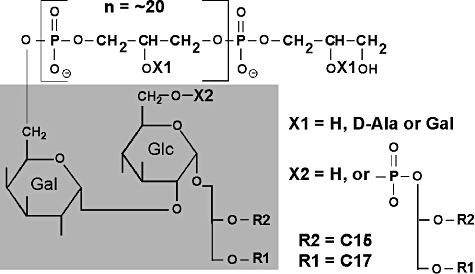
Chemical structure of *L. monocytogenes* LTA. *L. monocytogenes* LTA is a linear polyglycerolphosphate polymer attached to the membrane by the glycolipid Gal-Glc-DAG. The free hydroxyl group of the glycerolphosphate units (X1) can be esterified with d-alanine (d-Ala) or glycosylated with galactose (Gal) and the glucose moiety of Gal-Glc-DAG can be lipidated at position 6 with a phosphatidyl group (X2). The most abundant fatty acids in the glycolipid and the phosphatidyl substituent are C17 (R1) and C15 (R2) anteiso-branched fatty acids (Hether and Jackson, 1983; Uchikawa *et al*., 1986; Fischer *et al*., 1990).

The cell wall envelope of Gram-positive bacteria has been an area of active research for decades. By studying its assembly not only essential functions for bacterial growth and physiology but also important aspects of host pathogen interactions have been uncovered, and studies on the Gram-positive cell wall envelope have gained increased attention in the field of bacterial pathogens. A typical Gram-positive envelope is composed of peptidoglycan, proteins, often capsular polysaccharides and secondary wall polymers, which include wall teichoic acid (WTA), a polymer covalently linked to peptidoglycan, and lipoteichoic acid (LTA), a polymer tethered by a lipid anchor to the bacterial membrane (Fischer, 1988; Navarre and Schneewind, 1999). The structure of LTA varies between organisms (Fischer, 1988; Weidenmaier and Peschel, 2008); one of the best characterized structure is a polymer with an un-branched 1-3-linked glycerolphosphate chain attached to a membrane glycolipid as for instance found in *Bacillus subtilis*, *Staphylococcus aureus*, *Enterococcus faecalis*, Group A and B Streptococcus and *Listeria monocytogenes* (Fischer, 1990). Glycerolphosphate subunits can be substituted with glycosyl residues and/or d-alanine esters, which significantly contribute to cationic peptide resistance in Gram-positive bacteria (Fischer, 1990; Peschel *et al*., 1999). In *L. monocytogenes*, the polyglycerolphosphate LTA backbone is substituted with both d-alanines and α-galactosyl residues and linked to the bacterial membrane via glycolipids Gal(α1-2)Glc(α1-3)-diacylglycerol (Gal-Glc-DAG) or Gal(α1-2)Ptd-6Glc(α1-3)DAG (Gal-Ptd-6Glc-DAG), in which the glucose moiety is lipidated at position 6 with a phosphatidyl (Ptd) group (Hether and Jackson, 1983; Uchikawa *et al*., 1986; Fischer *et al*., 1990) (Fig. 1). Despite this thorough chemical analysis, the exact function of LTA is not known.

The recent identification of enzymes responsible for glycolipid and LTA backbone synthesis allowed a phenotypic characterization of strains that are deficient in LTA synthesis or produce LTA of an altered structure. The enzyme responsible for polyglycerolphosphate backbone chain formation has been discovered recently in *S. aureus* and named LtaS for LTA synthase (Gründling and Schneewind, 2007a). The same and two subsequent studies on *S. aureus* and *B. subtilis* revealed that LTA is important for normal growth and observed morphological alterations indicate a crucial role of LTA in the cell division process and the sporulation process in *B. subtilis* (Oku *et al*., 2009; Schirner *et al*., 2009).

Enzymes involved in the synthesis of glycolipids and lipid anchor for LTA have been characterized in several Gram-positive bacteria. In *S. aureus* and *B. subtilis* the enzyme YpfP (also called Ugt) is a processive glycosyltransferase, which synthesizes Glc(β1-6)Glc(β1-3)DAG (DiGlc-DAG) by the sequential addition of two glucose moieties onto diacylglycerol (DAG) using UDP-glucose as the substrate (Jorasch *et al*., 1998; 2000; Kiriukhin *et al*., 2001). On the other hand, in *E. faecalis* and *Streptococcus agalactiae* two separate enzymes are necessary for the synthesis of Glc(α1-2)Glc(β1-3)DAG (DiGlc-DAG) (Doran *et al*., 2005; Theilacker *et al*., 2009). The glycosyltransferase responsible for the addition of the second glucose moiety has been characterized in both organisms and renamed BgsA (EF2891 in strain V583) for biofilm-associated glycolipid synthesis and IagA (Gbs0682 in strain NEM316) for invasion-associated gene, respectively, to denote observed phenotypes and defects of deletion strains (Doran *et al*., 2005; Theilacker *et al*., 2009). It should be noted that phenotypes observed in strains mutated in glycolipid synthesis genes may not necessarily be due to the lack of these membrane lipids as LTA structure and production are also affected in their absence (Fedtke *et al*., 2007; Gründling and Schneewind, 2007b).

Little is known about LTA and glycolipid synthesis in the Gram-positive pathogen *L. monocytogenes*. Only the function of Dlt proteins, which incorporate d-alanines into LTA, has been investigated and it was found that this modification is important for bacterial adhesion to eukaryotic cells and virulence of *L. monocytogenes* in the mouse model of infection (Abachin *et al*., 2002). In addition, it has been reported that the *L. monocytogenes* internalin B protein (InlB), a non-covalently attached cell surface protein required for entry into various host cells, binds to LTA and is retained at the bacterial surface in this manner (Braun *et al*., 1997; 1998; Jonquieres *et al*., 1999). Thus, LTA directly and indirectly has important roles in bacterial physiology and virulence.

Here, we used a bioinformatics approach to identify *L. monocytogenes* genes required for glycolipid and LTA polyglycerolphosphate backbone synthesis. Using a combination of molecular biology and mass spectrometry approaches to characterize glycolipids and LTA synthesized in wild-type and mutant strains, we show that the previously uncharacterized *L. monocytogenes* genes *lmo2555* and *lmo2554* encode glycolipid synthesis enzymes, and renamed them LafA and LafB for LTA anchor formation proteins A and B. Two proteins, Lmo0927 and Lmo0644, with similarity to the *S. aureus* LTA synthase LtaS are involved in LTA backbone synthesis but they have clearly distinct enzymatic functions within the cell. Inactivation of Lmo0927 leads to the absence of LTA on the bacterial surface, a severe growth defect at elevated temperatures and morphological changes underscoring the importance of LTA for cellular functions in the Gram-positive pathogen *L. monocytogenes.*

## Results

### Identification of potential glycolipid and LTA synthesis gene in *L. monocytogenes*

**Fig. 2:**
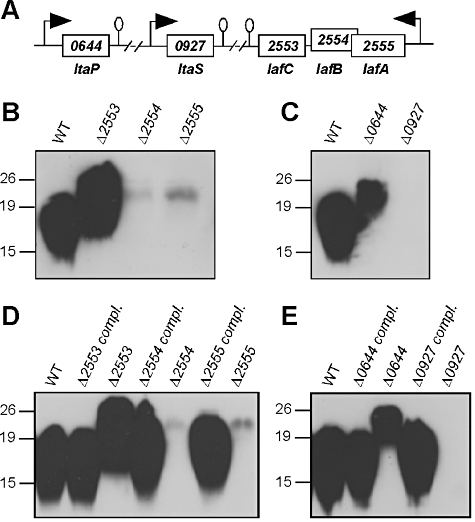
LTA production in wild-type and mutant *L. monocytogenes* strains. A. Operon structure of *L. monocytogenes* genes involved in glycolipid and LTA formation with direction of transcription indicated by the arrows and predicted terminators shown by hairpin loops. B–E. Western blot detection of cell wall-associated LTA in wild-type, deletion and complementation strains: (B) 10403S (WT), 10403SΔ*lmo2553* (Δ*2553*), 10403SΔ*lmo2554* (Δ*2554*) and 10403SΔ*lmo2555* (Δ*2555*); (C) 10403S (WT), 10403SΔ*lmo0644* (Δ*0644*) and 10403SΔ*lmo0927* (Δ*0927*); (D) 10403S pHPL3 (WT), 10403SΔ*lmo2553* pHPL3-*lmo2553* (Δ*2553 compl.*), 10403SΔ*lmo2553* pHPL3 (Δ*2553*), 10403SΔ*lmo2554* pHPL3-*lmo2554* (Δ*2554 compl.*), 10403SΔ*lmo2554* pHPL3 (Δ*2554*), 10403SΔ*lmo2555* pPL3-*lmo2555* (Δ*2555 compl.*) and 10403SΔ*lmo2555* pPL3 (Δ*2555*); (E) 10403S pPL3 (WT), 10403SΔ*lmo0644* pPL3-*lmo0644* (Δ*0644 compl.*), 10403SΔ*lmo0644* pPL3 (Δ*0644*), 10403SΔ*lmo0927* pPL3-*lmo0927* (Δ*0927 compl.*) and 10403SΔ*lmo0927* pPL3 (Δ*0927*). Positions of protein standards (in kDa) are shown on the left.

To begin to understand the function(s) of LTA in the Gram-positive pathogen *L. monocytogenes*, we used a bioinformatics approach to identify putative glycolipid and LTA synthesis enzymes in this organism. Since the LTA glycolipid anchor in *L. monocytogenes* consists of Gal-Glc-DAG (Hether and Jackson, 1983; Uchikawa *et al*., 1986; Fischer *et al*., 1990) presumably two distinct glycosyltransferases are required for its synthesis similar to that observed for *E. faecalis* and *S. agalactiae.* In *E. faecalis* and *S. agalactiae* the glycosyltransferases responsible for the addition of the terminal glucose moiety have been identified as IagA (Gbs0682 in strain NEM316) and BgsA (EF2891 in strain V583) and in both cases a second putative glycosyltransferase, Gbs0683 and EF2890, is encoded immediately upstream. These second proteins show high similarity to the characterized *Acholeplasma laidlawii* 1,2-diacylglycerol 3-glucosyltransferase (EC 2.4.1.157) (Berg *et al*., 2001) with *E*-values of 3e-53 and 2e-54. While experimental evidence is lacking, this suggests that Gbs0683 and EF2890 are responsible for Glc-DAG generation. We used the *S. agalactiae* proteins IagA (Gbs0682) and Gbs0683 as query sequences in blast searches (Altschul *et al*., 1997) against the *L. monocytogenes* EGD-e genome (Glaser *et al*., 2001). This identified the *L. monocytogenes* proteins Lmo2554 (*E*-value of 6e-20 to IagA) and Lmo2555 (*E*-value of 2e-76 to Gbs0683) as the closest homologues. Using the respective *E. faecalis* proteins in blast searches the same two *L. monocytogenes* proteins were identified with similar *E*-values of 2e-22 and 3e-83. Taken together, this suggests that Lmo2555 and Lmo02554 could encode UDP-glucose- and UDP-galactose-specific glycosyltransferases responsible for Glc-DAG and Gal-Glc-DAG synthesis respectively. The coding sequences of *lmo2555* and *lmo2554* overlap by eight bases and the operon is likely to contain a third gene, *lmo2553*, predicted to encode an integral membrane protein (Fig. 2A). The *S. aureus* enzyme LtaS, which is responsible for LTA polyglycerolphosphate backbone synthesis, was recently identified (Gründling and Schneewind, 2007a). Two proteins with high degree of similarity to the staphylococcal LtaS enzyme, Lmo0927 (*E*-value 0.0) and Lmo0644 (*E*-value 1e-58), are encoded in *Listeria* genomes (Fig. 2A).

To study the requirement of *lmo2555*, *lmo2554*, *lmo2553*, *lmo0927* and *lmo0644* for glycolipid production, LTA synthesis and bacterial physiology, these genes were inactivated in the *L. monocytogenes* 1/2a strain 10403S. Unmarked in-frame deletions were created by allelic exchange and all gene deletions were confirmed by PCR. With the exception of strain 10403SΔ*lmo0927*, which only grew well at 30°C (discussed below), all deletion strains had similar doubling times as compared with the parental 10403S strain (data not shown).

### Inactivation of predicted *L. monocytogenes* glycolipid and LTA synthases affects LTA production and structure

Initially we set out to assess if *lmo2555*, *lmo2554*, *lmo2553*, *lmo0927* and *lmo0644* contribute to LTA synthesis in *L. monocytogenes* by analysing LTA production in wild-type and deletion strains. In the case of *S. aureus*, inactivation of enzymes involved in glycolipid and LTA backbone synthesis leads to structural changes in LTA, which can be readily visualized by Western blot analysis using a polyglycerolphosphate-specific LTA antibody (Gründling and Schneewind, 2007b). Wild-type *L. monocytogenes* 104030S and deletion strains were grown overnight at 37°C with exception of strain 10403SΔ*lmo0927*, which was cultivated for 2 days at 37°C, and samples were prepared for Western blot analysis of cell wall associated LTA as described under *Experimental procedures*. Inactivation of Lmo2554 and Lmo2555 led to a drastic reduction in the total amount of LTA produced, while deletion of *lmo2553* led to the production of LTA with retarded mobility (Fig. 2B). A similar mobility shift was observed upon inactivation of Lmo0644, while no LTA-specific signal could be detected for strain 10403SΔ*lmo0927* (Fig. 2C). In addition, the amount of released LTA was analysed by the same Western blot method, but only minimal amounts could be detected in the culture supernatant of *lmo2555*, *lmo2554* and *lmo2553* deletion strains (data not shown). To confirm that observed phenotypes were solely due to deletion of the respective gene, complementation vectors were constructed and introduced into appropriate deletion strains. Genes *lmo0644*, *lmo0927* and *lmo2555* were cloned under their native promoter into the *L. monocytogenes* single-site integration vector pPL3, while *lmo2553* and *lmo2554* were cloned into vector pHPL3 under control of the hyper-spac promoter. As shown in Fig. 2D and E, the observed alterations in LTA production could be complemented, confirming that differences in LTA synthesis are due to inactivation of the respective gene. In summary, these results indicate that all *L.* monocytogenes proteins identified by our bioinformatics approach are indeed involved in LTA synthesis. The complete absence of LTA in strain 10403SΔ*lmo0927* suggests that Lmo0927 is responsible for the synthesis of the polyglycerolphosphate backbone chain, while Lmo0644 seems to have an accessory function.

### Lmo2555 and Lmo2554 are glycosyltransferases responsible for Glc-DAG and Gal-Glc-DAG production

**Fig. 3:**
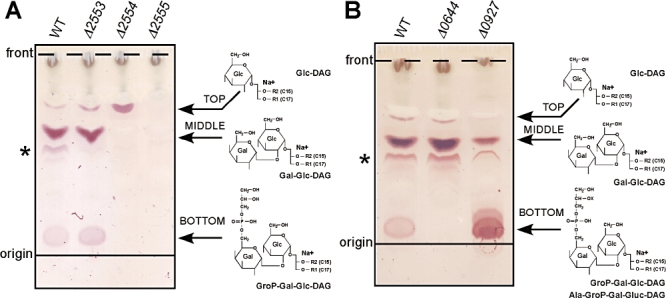
Glycolipid production in wild-type and mutant *L. monocytogenes* strains. Total membrane lipids were isolated from wild-type and deletion strains grown overnight at 30°C. Five hundred micrograms of lipids were separated by TLC and glycolipids visualized with α-naphthol/sulphuric acid. (A) 10403S (WT), 10403SΔ*lmo2553* (Δ*2553*), 10403SΔ*lmo2554* (Δ*2554*) and 10403SΔ*lmo2555* (Δ*2555*); (B) 10403S (WT), 10403SΔ*lmo0644* (Δ*0644*) and 10403SΔ*lmo0927* (Δ*0927*). Solid and dashed lines indicate the positions of origin and solvent front respectively. Top, middle and bottom glycolipid bands are indicated by arrows and structures, as identified in this study, are shown on the right. The glycolipid of unknown structure is marked with an asterisk.

**Fig. 4:**
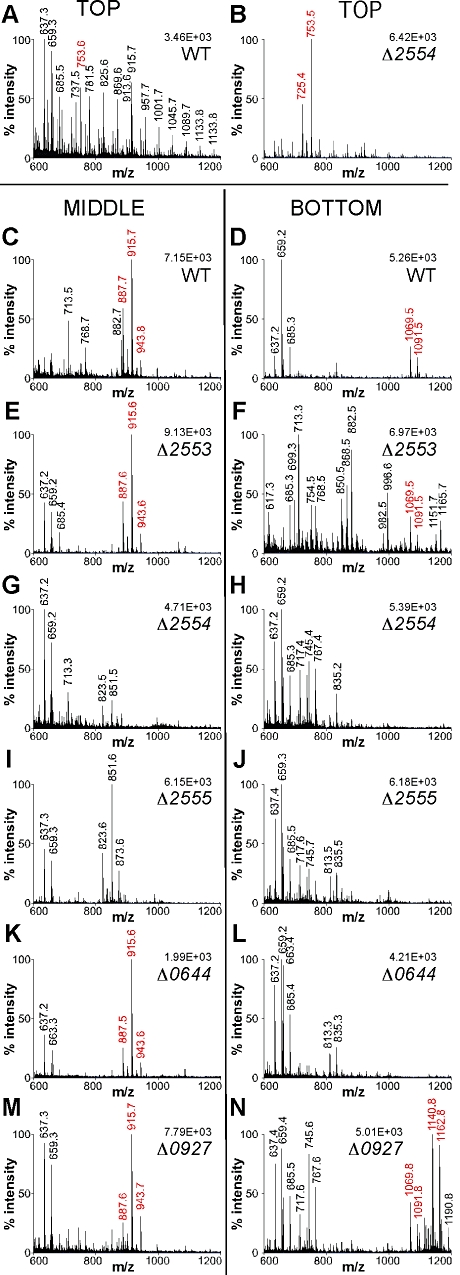
MALDI-TOF analysis of glycolipids produced by wild-type and mutant *L. monocytogenes* strains. Lipids from areas containing glycolipids labelled top, middle and bottom in Fig. 3 were further purified and subjected to MALDI-TOF mass spectrometry. Spectra are shown for (A) WT top band, (B) Δ*2554* top band, (C and D) WT, (E and F) Δ*2553*, (G and H) Δ*2554*, (I and J) Δ*2555*, (K and L) Δ*0644* and (M and N) Δ*0927* middle and bottom bands respectively. m/z signals on the *x*-axes are given in percentage (*y*-axes) of maximal value shown on the top left. Observed masses corresponding to predicted masses of glycolipids are highlighted in red.

Alterations in glycolipid synthesis will affect LTA structure and production (Gründling and Schneewind, 2007b). To correlate observed alterations in LTA production with changes in glycolipid formation in the different *L. monocytogenes* deletion strains, production of these membrane lipids was further analysed. Previous studies have identified the following glycolipids in membranes of *Listeria* spp.: Glc-DAG, Gal-Glc-DAG and glycolipids with the proposed structure of GroP-Gal-Glc-DAG (Gal-Glc-DAG with one glycerolphosphate subunit GroP) and the d-alanine-esterified derivative D-Ala-GroP-Gal-Glc-DAG (Fischer and Leopold, 1999). Bioinformatic analysis suggested that proteins encoded in the *lmo2555–lmo2553* operon are directly involved in glycolipid formation*.* For glycolipid analysis, wild-type, *lmo2553*, *lmo2554* and *lmo2555* deletion strains were grown overnight at 30°C, total membrane lipids isolated and separated by thin-layer chromatography (TLC). TLCs were developed with α-naphthol and sulphuric acid to visualize sugar-containing lipids. Four major glycolipid bands were detected in wild-type *L. monocytogenes* cells (Fig. 3A) and as shown in Fig. 3A, *lmo2555–lmo2553* deletion strains showed differences in the pattern of glycolipids as compared with the wild-type strain. Using matrix-assisted laser desorption/ionization (MALDI) mass spectrometry, we were able to provide structural information for glycolipids within three main bands labelled top, middle and bottom in Fig. 3. Obtained masses were consistent with expected masses for sodium adducts of Glc-DAG (753.6 m/z – top band; Fig. 4A), Gal-Glc-DAG (915.7 m/z – middle band; Fig. 4C) and GroP-Gal-Glc-DAG (1069.5 m/z – bottom band; Fig. 4D) with C17 and C15 fatty acid side-chains. Calculated absolute masses for glycolipids and experimentally observed masses are summarized in Table 1. The predominant masses for lipids with C17 and C15 fatty acid chains are consistent with previous findings that anteiso-branched C17 and C15 fatty acid at C1 and C2 positions, respectively, are the most abundant fatty acids in *Listeria* lipids (Kosaric and Carroll, 1971; Fischer and Leopold, 1999). In addition, we also observed masses, which are consistent with glycolipids with C15/C15 or C17/C17 fatty acid chains or disodium adducts (replacement of H^+^ with Na^+^) (Table 1). Only small amounts of Glc-DAG accumulated in wild-type cells (Fig. 3A) and as seen in Fig. 4A the observed mass signal of 753.6 m/z for this lipid was weak as compared with background signals but the signal was specific (background peaks were separated from each other by 44 mass units). Deletion of *lmo2553*, predicted to encode an integral membrane protein, resulted in small but reproducible changes in the glycolipid profile with reduction of a glycolipid of unknown structure, indicated by an asterisk in Fig. 3A. Deletion of *lmo2555* led to a complete absence of glycolipids and deletion of *lmo2554* led to the accumulation of Glc-DAG (Fig. 3A). Glycolipid structure as well as presence and absence of Gal-Glc-DAG (middle band) and GroP-Gal-Glc-DAG (bottom band) was confirmed by MALDI mass spectrometry (Fig. 4B and E–J, Table 1). We had difficulties detecting a signal above background for the top glycolipid band. Only in strain 10403SΔ*lmo2554*, in which this lipid accumulated to significant levels, a clear m/z signal of 753.5 was obtained as expected for a lipid with the structure Glc-DAG (Fig. 4B). These results are consistent with a function of Lmo2555 and Lmo2554 as glycosyltransferase responsible for the formation of Glc-DAG and Gal-Glc-DAG respectively. The exact function of Lmo2553 remains unknown; nevertheless we propose to rename proteins encoded in the *lmo2555–lmo2553* operon, LafA (Lmo2555), LafB (Lmo2554) and LafC (Lmo2553) for LTA anchor formation proteins A to C.

**Table 1:** Summary of TLC and MALDI-TOF data of glycolipids produced by wild-type and mutant *L. monocytogenes* strains.

Lipid (fatty acid chain length) Formula – calculated mass	WT	Δ*2553*	Δ*2554*	Δ*2555*	Δ*0644*	Δ*0927*
Top band	X	X	X	Not observed	X	X
Glc-DAG (C17, C15)						
C41H78Na1O10 – 753.5	753.6		753.5			
Glc-DAG (C15, C15)						
C39H74Na1O10 – 725.5	725.4					
Middle band	X	X	Not observed	Not observed	X	X
Gal-Glc-DAG (C17, C15)						
C47H88Na1O15 – 915.6	915.7	915.6			915.6	915.7
Gal-Glc-DAG (C15, C15)						
C45H84Na1O15 – 887.6	887.6	887.6			887.6	887.6
Gal-Glc-DAG (C17, C17)						
C49H92Na1O15 – 943.6	943.8	943.6			943.6	943.7
Bottom band	X	X	Not observed	Not observed	Not observed	X
GroP-Gal-Glc-DAG (C17, C15)						
C50H95Na1O20P1 – 1069.6	1069.5	1069.5				1069.8
GroP-Gal-Glc-DAG (C17, C15)						
C50H94Na2O20P1 – 1091.6 (disodium adduct)	1091.5	1091.5				1091.8
Ala-GroP-Gal-Glc-DAG (C17, C15)	Not observed	Not observed	Not observed	Not observed	Not observed	X
C53H100 N1Na1O21P1 – 1140.6						1140.8
Ala-GroP-Gal-Glc-DAG (C17, C15)						
C53H99 N1Na2O21P1 – 1162.6 (disodium adduct)						1162.8

Presence or absence of glycolipids in different *L. monocytogenes* strains shown on top is denoted with ‘X’ when present and ‘Not observed’ when absent. Abbreviations for glycolipids in top, middle and bottom bands as indicated in Fig. 3 are shown in the left column with fatty acid chain length given in parenthesis along with molecular formula and calculated absolute mass of sodium adducts or disodium adducts (minus one proton).

### Lmo0644 functions as an LTA primase

Based on the accumulation of glycolipids with a single glycerolphosphate in some Gram-positive bacteria such as GroP-Gal-Glc-DAG in *L. monocytogenes*, a two-enzyme system for LTA synthesis has been proposed, whereby an LTA primase initiates LTA synthesis by the transfer of the first glycerolphosphate subunit onto the glycolipid and a second enzyme, an LTA synthase, extends the chain to form the polyglycerolphosphate backbone (Fischer, 1990). Lmo0927 is likely to be the LTA synthase, as in its absence no LTA could be detected on the bacterial surface (Fig. 2C). In the presence of an LTA primase, one would expect that even in the absence of the LTA synthase (Lmo0927) production of GroP-Gal-Glc-DAG should occur. To test if Lmo0644 could serve as LTA primase, we determined the glycolipid profile of *lmo0644* and *lmo0927* deletion strains. In the absence of Lmo0644 no GroP-Gal-Glc-DAG (bottom band) could be detected, while in the absence of Lmo0927 GroP-Gal-Glc-DAG (bottom band) was produced and accumulated to higher levels as compared with a wild-type strain (Fig. 3B). MALDI mass spectrometry analysis confirmed the structure and presence or absence of Gal-Glc-DAG and GroP-Gal-Glc-DAG in respective deletion strains (Fig. 4K–N; Table 1). The m/z signal of 1069.8, expected for a sodium adduct of GroP-Gal-Glc-DAG with a calculated mass of 1069.6, was only observed in the presence of Lmo0644. In addition, upon deletion of *lmo0927*, which resulted in the accumulation of the bottom glycolipid band, an additional mass signal of 1040.8 was observed, which is consistent with the expected mass of 1040.6 for d-Ala-GroP-Gal-Glc-DAG, a d-Ala esterified derivative of GroP-Gal-Glc-DAG. These results are consistent with a model whereby Lmo0644 serves as LTA primase and Lmo0927 functions as LTA synthase and we propose to rename Lmo0644, LtaP, for LTA primase and Lmo0927, LtaS, for LTA synthase.

### LtaP (Lmo0644) and LtaS (Lmo0927) are processed in *L. monocytogenes*

**Fig. 5:**
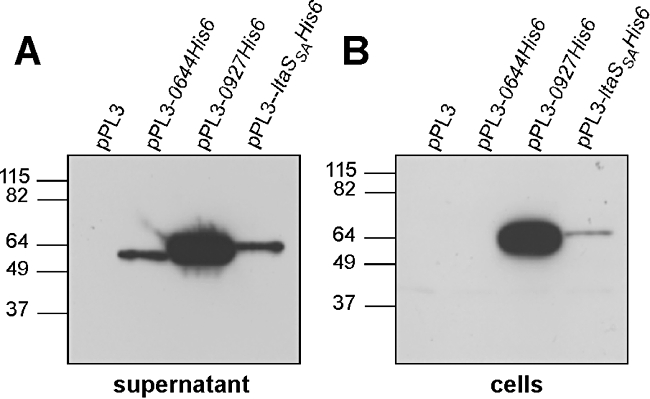
Production and processing of *L. monocytogenes* proteins Lmo0644 and Lmo0927. *L. monocytogenes* strains expressing C-terminally His-tagged versions of Lmo0644, Lmo0927 and the staphylococcal LtaS_SA_ control protein were grown overnight at 37°C and samples prepared for Western blot analysis as described under *Experimental procedures*. (A) Supernatant and (B) cell wall-associated protein samples were separated on 10% SDS polyacrylamide gels and tagged proteins detected with a His-tag-specific antibody. Samples were obtained from *L. monocytogenes* strains 10403S pPL3 (pPL3), 10403S pPL3-*lmo0644His6* (pPL3-*0644His6*), 10403S pPL3-*lmo0927His6* (pPL3-*0927His6*) and 10403S pPL3-*ltaS*_*SA*_*His6* (pPL3-*ltaS*_*SA*_*His6*). The positions of protein standards (in kDa) are indicated on the left. Predicted masses for his-tagged full-length and cleaved proteins are 75.3 and 50.2 kDa for LtaS_SA_, 75.6 and 49.5 kDa for Lmo0927, and 70.1 and approximately 48 kDa for Lmo0644.

In *S. aureus*, LtaS is efficiently processed and the extracellular enzymatic domain can be found in the culture supernatant as well as in the cell wall fraction (Lu *et al*., 2009). To test if *L. monocytogenes* LtaP (Lmo0644) and LtaS (Lmo0927) are processed similarly, localization and cleavage of these proteins was analysed. To this end, plasmids pPL3-*lmo644His6* and pPL3-*lmo927His6* were constructed for expression of C-terminally His-tagged LtaP and LtaS proteins under their native promoter and integrated into the chromosome of strain 10403S. We also introduce as controls the empty vector pPL3 and pPL3-*ltaS*_*SA*_*His6*, which encodes a C-terminally tagged version of the *S. aureus* LtaS protein under the control of its native promoter. Resulting strains 10403S pPL3, 10403S pPL3-*lmo644His6*, 10403S pPL3-*lmo927His6* and 10403S pPL3-*ltaS*_*SA*_*His6* were grown overnight at 37°C and cell-associated and supernatant protein samples were prepared for Western blot analysis as described in the *Experimental procedure* section and tagged proteins were detected with a His-tag-specific antibody. As can be seen in Fig. 5A, *L. monocytogenes* LtaP and LtaS, and the *S. aureus* LtaS_SA_ protein could be detected in culture supernatant, indicating that all proteins were cleaved in *L. monocytogenes*. Processed forms of the *L. monocytogenes* LTA synthase (Lmo927) and the *S. aureus* control protein were also detected in the cell wall-associated fraction (Fig. 5B). We were not able to detect any full-length proteins in *L. monocytogenes*, while our previously published results have shown that small amounts of full-length LtaS_SA_ protein can be detected in *S. aureus* using an identical sample preparation method (Lu *et al*., 2009). Taken together, these results show that both proteins, LtaP and LtaS, are processed in *L. monocytogenes* and suggest that simply a difference in enzyme processing cannot explain the difference in enzyme function between an LTA primase and an LTA synthase.

### Inactivation of the LTA synthase LtaS (Lmo0927) severely affects growth and morphology of *L. monocytogenes*

**Fig. 6:**
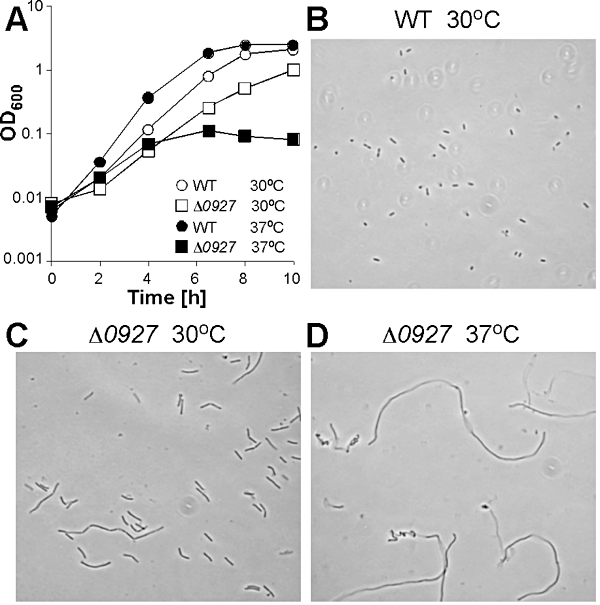
Growth and morphology of wild-type 10403S and 10403SΔ*lmo0927 L. monocytogenes* strains. A. Bacterial growth curves. Overnight cultures of wild-type 10403S (WT) and 10403SΔ*lmo927* (Δ*0927*) strains were diluted into fresh BHI medium and cultures incubated at 30°C or 37°C. OD_600_ values were determined at timed intervals and plotted. B–D. Phase-contrast microscopy images of (B) 10403S (WT) and (C and D) 10403SΔ*lmo0927* (Δ*0927*) strains grown for 8.5 h at the indicated temperature.

**Fig. 7:**
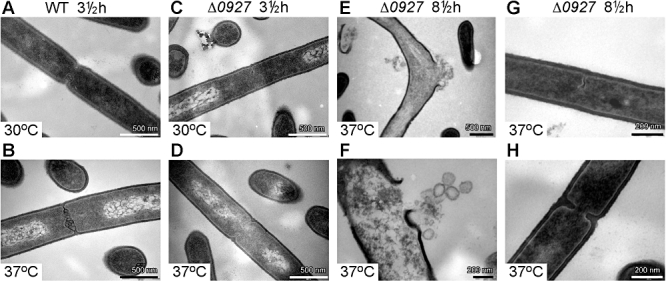
Transmission electron microscopy (TEM) images of wild-type 10403S and 10403SΔ*lmo0927 L. monocytogenes* strains. Overnight cultures of wild-type 10403S (WT) and 10403SΔ*lmo927* (Δ*0927*) strains were back-diluted and grown for the indicated time at 30°C or 37°C. Bacteria were fixed and prepared for TEM as described under *Experimental procedures* and representative images are shown: WT grown for 3.5 h at (A) 30°C and (B) 37°C; Δ*0927* grown for 3.5 h at (C) 30°C and (D) 37°C; (E–H) Δ*0927* grown for 8.5 h at 37°C. Images were taken at (A–D) 49 000×; (E) 30 000×; (F) 68 000×; (G and H) 98 000× magnification and scale bars are shown.

LtaS is required for growth of *S. aureus* under standard laboratory growth conditions (tryptic soya broth medium at 37°C) (Gründling and Schneewind, 2007a). An LtaS deletion strain is viable at 30°C when grown in the present of at least 1% NaCl and at 37°C when bacteria are cultured in the presence of 7.5% NaCl or 40% sucrose (Oku *et al*., 2009). However, *S. aureus* cells have severe morphological defects in the absence of LTA (Gründling and Schneewind, 2007a; Oku *et al*., 2009). *L. monocytogenes* is apparently able to grow in the absence of LTA since we obtained a strain with a clean deletion in *lmo0927* (see Fig. 2C). However, during the strain construction, we observed that an *lmo0927* deletion strain could only be obtained when bacteria were plated and maintained at 30°C (and not at 37°C) during the final step of the allelic exchange procedure. A more detail growth analysis revealed that strain 10403SΔ*lmo0927* had already at 30°C a reduced colony size (data not shown) and reduced growth rate as compared with a wild-type strain (Fig. 6A) and growth ceased at 37°C (Fig. 6A). This severe growth defect at 37°C was corroborated by a more than 5-log reduction in the plating efficiency at 37°C [8.7 ± 0.6 × 10^3^ colony-forming units (cfu) ml^−1^] as compared with 30°C (2.8 ± 0.5 × 10^9^ cfu ml^−1^). In addition, strain 10403SΔ*lmo0927* formed short chains at 30°C and chain length increased upon incubation at 37°C (Fig. 6B and D). Transmission electron microscopy (TEM) analysis showed that strain 10403SΔ*lmo0927* had a pronounced defect in division septum formation (Fig. 7). As already seen previously in electron microscopy images of *L. monocytogenes* (Edwards and Stevens, 1963), ample membrane material in the form of ‘bubbles’ was observed at the division sites of wild-type cells (Fig. 7A and B). Such membrane bubbles were never observed in 10403SΔ*lmo0927*, regardless of the growth temperature (Fig. 7C–H). The complete absence of membranous material at division sites in the *lmo0927* deletion strain was more frequent at the non-permissive growth temperature and 8.5 h after the temperature shift actual cell lysis was observed in several cells (Fig. 7E and F). In the few instances where a clear division site was observed in strain 10403SΔ*lmo0927*, these septa looked abnormal (Fig. 7H). In summary, these data show that LTA plays a crucial role in the cell division process in another Gram-positive pathogen, the rod-shaped bacterium *L. monocytogenes*.

## Discussion

**Fig. 8:**
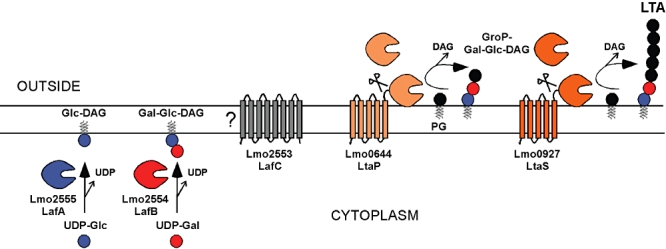
Model for glycolipid and LTA synthesis in *L. monocytogenes*. The cytoplasmic glycosyltransferases Lmo2555 (LafA, LTA anchor formation protein A; shown in blue) and Lmo2554 (LafB; shown in red) synthesize Glc-DAG and Gal-Glc-DAG, respectively, presumably using nucleotide-activated sugars UDP-Glc and UDP-Gal as substrates. Lmo2553 (LafC, shown in grey) is a membrane protein of unknown function and likely acts downstream of LafA and LafB in the glycolipid synthesis pathway. *L. monocytogenes* uses a two-enzyme system for the subsequent polyglycerolphosphate LTA chain formation. The LTA primase Lmo0644 (LtaP, shown in light orange) transfers the initial glycerolphosphate (black circle) derived from phosphatidylglycerol (PG) onto Gal-Glc-DAG, resulting in the production of GroP-Gal-Glc-DAG. The LTA synthase Lmo0927 (LtaS, shown in orange) then transfers additional glycerolphosphate residues onto GroP-Gal-Glc-DAG, thereby forming the polyglycerolphosphate backbone chain of LTA.

In this study, we have identified and characterized *L. monocytogenes* proteins required for glycolipid anchor and LTA backbone synthesis and a summary model for their function is shown in Fig. 8.

Based on the structural relation of glycolipids with a single glycerolphosphate subunit (GroP-glycolipids) and their parallels in occurrence with LTA, it has been suggested that GroP-glycolipids are intermediates in the LTA synthesis pathway (Fischer *et al*., 1978; 1990; Fischer, 1981). This led to the proposal that LTA is synthesized by two enzymes: an LTA primase, which produces the GroP-glycolipid intermediate and an LTA synthase, which extends the polyglycerolphosphate backbone chain on this intermediate (Fischer *et al*., 1978; 1990; Fischer, 1981). This and alternative models for polyglycerolphosphate chain synthesis and extension have been proposed and recently reviewed by Rahman *et al*. (2009). Here, we provide evidence that *L. monocytogenes* uses a two-enzyme system for LTA synthesis and show for the first time distinct enzymatic functions for two LtaS paralogues within the cell. Based on the results presented in Figs 2–4, we propose that LtaP (Lmo0644) acts as an LTA primase and produces GroP-glycolipids and LtaS (Lmo0927) functions as LTA synthase and generates the polyglycerolphophate backbone. However, even in the absence of LtaP (*lmo0644* mutant), the *L. monocytogenes* LtaS enzyme can produce a polyglycerolphosphate polymer (Fig. 2C) and we speculate that LtaS can initiate LTA synthesis on both glycolipids and in the absence of this class of lipids directly on DAG or PG. This notion is supported by the observed LTA profile of an *ltaP/lafA* (*lmo0644/lmo2555*) double mutant (Fig. S1A). In the absence of both, glycolipids (*lmo2555* mutant) and the LTA primase (*lmo0644* mutant), LTA is produced albeit at reduced levels compared with a strain, which only lacks the LTA primase. This indicates that in the absence of LtaP, LtaS can use glycolipids as an anchor and in the absence of both, LtaP and glycolipids, DAG or PG is used. (It should be noted that the observed LTA alterations in the double mutant could be complemented to the levels of the individual single mutants by introduction of the respective complementation vector pPL3-*lmo0644* or pPL3-*lmo2555*; Fig. S1A).

In contrast to *L. monocytogenes*, *S. aureus* apparently synthesizes LTA with a single enzyme and depletion of LtaS_SA_ does not lead to an accumulation of GroP-glycolipids (M. Wörmann, unpubl. results). The biological significance why some bacteria use a one-enzyme and other bacteria use a multienzyme system for LTA synthesis is not clear at this point, especially considering that an *L. monocytogenes ltaP* mutant still synthesizes LTA and does not show a growth defect in broth culture. While it is not clear why different Gram-positive bacteria use one or multiple enzymes for LTA synthesis, in general there seems to be a correlation between the number of genome-encoded LtaS-like proteins and bacterial shape; coccoid *Staphylococcus* spp., *Streptococcus* spp. (with exception of *S. pneumoniae*, which does not produce a polyglycerolphosphate-type LTA and does not encode an LtaS-like protein) and *Lactococcus lactis* strains encode one LtaS protein; ellipsoid-shaped *E. faecalis* strains, rod-shaped *Listeria* spp. and with a few exceptions rod-shaped *Lactobacillus* spp. encode two proteins; and the majority of *Bacillus* spp., rod-shaped bacteria with a more complex developmental cycle, encode multiple LtaS-like proteins. *B. subtilis* contains four LtaS paralogues (Gründling and Schneewind, 2007a) and based on published results, it was suggested that the four *B. subtilis* LtaS paralogues have partially different functions and individual or a combination of these proteins is particular important for proper septum formation during cell division (YlfE) or the sporulation process (YflE and YqgS) (Schirner *et al*., 2009).

Of the five deletion strains constructed in this study, only deletion of *ltaS*, which caused a complete absence of LTA (Fig. 2C), had a marked effect on bacterial growth (Figs 6 and 7). The morphological changes observed in *L. monocytogenes* in the absence of LTA were very similar to those observed in a *B. subtilis yflE* mutant and seemed to be primarily due to cell division defects (Figs 6 and 7). During the cell division process, a large amount of new membrane and other cell wall material needs to be synthesized at the division site. In wild-type 10403S *L. monocytogenes* cells, ample membrane material in the form of ‘bubbles’ is observed at the division site (Fig. 7). A striking difference between wild-type and *lmo0927* mutant cells is the lack of such large amounts of membranous material at the division site, even at temperatures that are permissive for bacterial growth (Fig. 7). LTA and membrane lipid synthesis are intimately connected, as glycolipids are used as the LTA anchor and the glycerolphosphate subunits of the LTA backbone are likely derived from the headgroup of the membrane lipid phosphatidylglycerol (PG) (Fig. 8). Based on results obtained in this and previous studies (Gründling and Schneewind, 2007a; Oku *et al*., 2009; Schirner *et al*., 2009) a unifying scheme has emerged, in which LTA plays an especially important function in the cell division process of Gram-positive bacteria, although the mechanism remains unclear. It has been suggested that LTA ensures that sufficient Mg^2+^ is available for enzymes acting at the leading edge of the invaginating division septum to function (Schirner *et al*., 2009). Alternatively, LTA could directly or indirectly contribute to specific membrane properties, which are important for the formation of division septa and the cell division process.

In this study, we further show that LafA (Lmo2555) and LafB (Lmo2554) are necessary for the production of Glc-DAG and Gal-Glc-DAG respectively (Fig. 3A). These enzymes likely use the nucleotide-activated sugars UDP-glucose and UDP-galactose as substrates (see model Fig. 8). A conserved catalytic EX_7_E signature sequence is present in both LafA (amino acids 293–301) and LafB (amino acids 252–260). This sequence is also found in the characterized glycosyltransferases alMGS and alDGS from *A. laidlawii*, which belong to the CAZy family 4 of glycosyltransferases (Campbell *et al*., 1997; Berg *et al*., 2001; Edman *et al*., 2003). In the absence of Gal-Glc-DAG, a profound reduction in the overall amount of LTA was observed (Fig. 2B). However, *lafA* or *lafB* mutants did not show a reduced growth rate in broth culture suggesting that small amounts of LTA are sufficient for bacterial growth under the conditions tested.

Inactivation of glycolipid-synthesizing enzymes in other Gram-positive bacteria can have different effects on the overall production of LTA. While inactivation of YpfP in *S. aureus* strains Newman and RN4220 leads to an overall increase in the amount of LTA synthesized and released into the culture supernatant (Kiriukhin *et al*., 2001; Gründling and Schneewind, 2007b), a drastic reduction in LTA has been reported for a *ypfP* mutant in the SA113 strain background (Fedtke *et al*., 2007), which is what we observed for *lafA* or *lafB* mutant *L. monocytogenes* strains. The simplest explanation for this reduction in LTA production is that enzyme(s), which are subsequently needed for the formation of the polyglycerolphosphate polymer, cannot efficiently initiate LTA synthesis in the absence of glycolipids. We suggest that in *L. monocytogenes* in the absence of glycolipids, neither LtaP nor LtaS can efficiently initiate polyglycerolphosphate LTA backbone synthesis. Therefore, LtaP and LtaS could either have a specific recognition site for glycolipids or alternatively special constraints (the OH-group used for backbone extension will be further removed from the membrane surface in glycolipids as compared with other lipids such as DAG or PG) could dictate which lipid can be used to initiate LTA synthesis. Structural information on the extracellular *S. aureus* LtaS or *B. subtilis* YflE domains did not provided any information on potential glycolipid binding sites (Lu *et al*., 2009; Schirner *et al*., 2009) and these sites might be embedded within the membrane portion of the protein, for which no structural information is available. For *S. aureus* strains, differences in LtaS protein sequence could explain observed phenotypic differences in *ypfP* mutants. However, the LtaS sequence does not vary between sequenced strains, with exception of strain RF122, which has one amino acid change. If not differences in LtaS protein sequence, differences in expression, post-translational modifications or differences in yet to be identified proteins involved in LTA synthesis could explain observed strain differences.

A third gene, *lafC* (*lmo2553*), predicted to code for an integral membrane protein with eight transmembrane helices is part of the *laf* operon. We show that LafC plays an accessory function in glycolipid and LTA synthesis as inactivation of LafC results in minor changes in the glycolipid profile (Fig. 3A) and production of LTA with a retarded mobility similar to that observed in an *ltaP* mutant (Fig. 2B and C). Since glycolipids Glc-DAG and Gal-Glc-DAG are produced in a *lafC* mutant, this protein probably acts downstream of LafA and LafB (see model in Fig. 8). During this study, we also created an *ltaP/lafC* (*lmo0644/lmo2553*) double mutant and respective control strains for complementation analysis. LTA analysis revealed that the strain lacking both, the LTA primase LtaP and LtaC, produced only small amounts of LTA, comparable to that of the *lafA* (*lmo2555*) mutant, which is unable to synthesize glycolipids (Fig. S1B). This result indicates that either LafC or LtaP is required presumably to modify the glycolipid in such a manner that it can be used by LtaS as the LTA anchor. In several *Listeria* spp., LTA is anchored to the membrane not only by Gal-Glc-DAG but also by a derivative in which the glucose moiety is lipidated at position 6 with a Ptd group (Fig. 1) (Uchikawa *et al*., 1986) and LafC could be involved in its synthesis. Such a modification may physically alter the presentation of the anchor thus affecting the ability of LtaS to synthesize LTA. However, additional work is needed to determine the exact molecular function of LafC and to establish the requirement of glycolipids and LTA during infection. Also, determining the precise function of LTA during the cell division process warrants further investigation.

## Experimental procedures

### Bacterial strains, plasmids and growth conditions

Bacterial strains and plasmids used in this study are listed in Table 2. *L. monocytogenes* mutants were constructed in strain 10403S, which is a streptomycin resistant isolate of the serotype 1/2a strain 10403 (Bishop and Hinrichs, 1987). *L. monocytogenes* strains were grown in brain heart infusion (BHI) medium at 30°C or 37°C as indicated and *Escherichia coli* strains were grown in Luria–Bertani (LB) medium at 37°C. When appropriate, the growth medium was supplemented with antibiotics as listed in Table 2.

**Table 2:** Bacterial strains used in this study.

Strain	Relevant features	Reference
*Escherichia coli* strains		
XL1 Blue	Cloning strain, TetR – ANG127	Stratagene
CLG190	Cloning strain, TetR – ANG1141	D. Boyd
SM10	*E. coli* strain used for conjugations; KanR – ANG618	Simon *et al*. (1983)
ANG124	JM109 pKSV7; allelic exchange vector; AmpR	Smith and Youngman (1992)
ANG243	XL1-Blue with *S. aureus* integration vector pCL55	Lee *et al*. (1991)
ANG583	XL1-Blue pCL55-*ltaS*_*SA*_*His6; S. aureus* LtaS (SAV0719) with C-terminal His6 tag; AmpR	This study
ANG1378	CLG190 pKSV7Δ*lmo0644*; AmpR	This study
ANG1379	XL1 Blue pKSV7Δ*lmo0927*; AmpR	This study
ANG1382	XL1 Blue pKSV7Δ*lmo2553*; AmpR	This study
ANG1384	XL1 Blue pKSV7Δ*lmo2554*; AmpR	This study
ANG1385	XL1 Blue pKSV7Δ*lmo2555*; AmpR	This study
DH-E898	XL1 Blue pPL3; *L. monocytogenes* integration vector; CamR – ANG1276	Gründling *et al*. (2004)
DH-E899	XL1 Blue pHPL3; *L. monocytogenes* integration vector with hyper-spac promoter; CamR – ANG1277	Gründling *et al*. (2004)
AJW1392	XL1 Blue pPL3-*lmo0644*; *lmo0644* under native promoter control; CamR	This study
AJW1393	XL1 Blue pPL3-*lmo0927*; *lmo0927* under native promoter control; CamR	This study
AJW1396	XL1 Blue pHPL3-*lmo2553*; *lmo2553* under hyper-spac promoter control; CamR	This study
AJW1397	XL1 Blue pHPL3-*lmo2554*; *lmo2554* under hyper-spac promoter control; CamR	This study
AJW1398	XL1 Blue pPL3-*lmo2555*; *lmo2555* under native promoter control; CamR	This study
ANG1399	XL1 Blue pPL3-*lmo0644His6*; Lmo0644 with C-terminal His-tag under native promoter control; CamR	This study
ANG1401	XL1 Blue pPL3-*lmo0927His6*; Lmo0927 with C-terminal His-tag under native promoter control; CamR	This study
ANG1406	XL1 Blue pPL3-*ltaS*_*SA*_*His6*; LtaS_SA_ with C-terminal His-tag under native promoter control; CamR	This study
ANG1456	SM10 pPL3; *E. coli* conjugation strain donor for plasmid pPL3, KanR, CamR	This study
ANG1459	SM10 pPL3-*lmo0927*; *E. coli* conjugation strain donor for plasmid pPL3-*lmo0927*, KanR, CamR	This study
*Listeria monocytogenes* strains		
10403S	StrepR – ANG1263	Bishop and Hinrichs (1987)
AJW1385	10403SΔ*lmo0644*; StrepR	This study
ANG1386	10403SΔ*lmo0927*; StrepR	This study
AJW1389	10403SΔ*lmo2553*; StrepR	This study
AJW1390	10403SΔ*lmo2554*; StrepR	This study
AJW1391	10403SΔ*lmo2555*; StrepR	This study
ANG1411	10403SΔ*lmo0927* pPL3; StrepR, CamR	This study
ANG1412	10403SΔ*lmo0927* pPL3-*lmo0927*; *lmo0927* complementation strain; StrepR, CamR	This study
AJW1413	10403S pPL3; StrepR, CamR	This study
AJW1414	10403S pHPL3; StrepR, CamR	This study
AJW1415	10403SΔ*lmo0644* pPL3-*lmo0644*; *lmo0644* complementation strain; StrepR, CamR	This study
AJW1416	10403SΔ*lmo0644* pPL3; StrepR, CamR	This study
AJW1417	10403SΔ*lmo2553* pHPL3-*lmo2553*; *lmo2553* complementation strain; StrepR, CamR	This study
AJW1418	10403SΔ*lmo2553* pHPL3; StrepR, CamR	This study
AJW1419	10403SΔ*lmo2554* pHPL3-*lmo2554*; *lmo2554* complementation strain; StrepR, CamR	This study
AJW1420	10403SΔ*lmo2554* pHPL3; StrepR, CamR	This study
AJW1421	10403SΔ*lmo2555* pPL3-*lmo2555*; *lmo2555* complementation strain; StrepR, CamR	This study
AJW1422	10403SΔ*lmo2555* pPL3; StrepR, CamR	This study
AJW1423	10403S pPL3-*lmo0644His6*; StrepR, CamR	This study
AJW1424	10403S pPL3-*lmo0927His6*; StrepR, CamR	This study
AJW1425	10403S pPL3-*ltaS*_*SA*_*His6*; StrepR, CamR	This study
AJW1496	10403S Δ*lmo0644*Δ*lmo2555*; StrepR	This study
AJW1497	10403S Δ*lmo0644*Δ*lmo2555* pPL3; StrepR, CamR	This study
AJW1498	10403S Δ*lmo0644*Δ*lmo2555* pPL3-*lmo0644*; *lmo0644* complementation strain; StrepR, CamR	This study
AJW1499	10403S Δ*lmo0644*Δ*lmo2555* pPL3-*lmo2555*; *lmo2555* complementation strain; StrepR, CamR	This study
AJW1501	10403S Δ*lmo0644*Δ*lmo2553*; StrepR	This study
AJW1502	10403S Δ*lmo0644*Δ*lmo2553* pPL3; StrepR, CamR	This study
AJW1503	10403S Δ*lmo0644*Δ*lmo2553* pPL3-*lmo0644*; *lmo0644* complementation strain; StrepR, CamR	This study
AJW1504	10403S Δ*lmo0644*Δ*lmo2553* pHPL3-*lmo2553*; *lmo2553* complementation strain; StrepR, CamR	This study
Other strains		
RN4220	Transformable *S. aureus* laboratory strain – ANG113	Kreiswirth *et al*. (1983)

Antibiotics were used at the following concentrations: for *E. coli* cultures: ampicillin (AmpR) 100 μg ml^−1^; kanamycin (KanR) 30 μg ml^−1^; tetracycline (TetR) 10 μg ml^−1^; for *L. monocytogenes* cultures: chloramphenicol (CamR) 7.5 or 10 μg ml^−1^; streptomycin 200 μg ml^−1^ (StrepR) for the conjugation experiment.

### Plasmid and strain construction

Pfu polymerase (Stratagene) was used for PCR amplification of DNA fragments subsequently used for cloning. Restriction enzymes were purchased from New England Biolabs and used according to the manufacturer's instructions. All plasmids were constructed initially in *E. coli* strains XL1-Blue or CLG190. Allelic exchange plasmids for the construction of strains 10403SΔ*lmo0644*, 10403SΔ*lmo0927*, 10403SΔ*lmo2553*, 10403SΔ*lmo2554* and 10403SΔ*lmo2555* containing an in-frame deletion in the respective gene were constructed by the two-step PCR SOE method (Horton *et al*., 1989) using primers listed in Table 3. Briefly, primers 5-KpnI-LMO0644, 5-int-LMO0644, 3-BamHI-LMO0644 and 3-int-LMO0644-10403S (for deletion of *lmo0644*); 5-KpnI-LMO0927, 5-int-LMO0927, 3-BamHI-LMO0927 and 3-int-LMO0927 (for deletion of *lmo0927*); 5-KpnI-LMO2553, 5-int-LMO2553, 3-BamHI-LMO2553 and 3-int-LMO2553 (for deletion of *lmo2553*); 5-KpnI-LMO2554, 5-int-LMO2554, 3-BamHI-LMO2554 and 3-int-LMO2554 (for deletion of *lmo2554*); 5-KpnI-LMO2555, 5-int-LMO2555, 3-BamHI-LMO2555 and 3-int-LMO2555 (for deletion of *lmo2555*) were used to amplify and fuse approximately 950 bp upstream and downstream regions of relevant genes using 10403S chromosomal DNA in PCR reactions. The resulting PCR products were digested with the restriction enzymes KpnI and BamHI and ligated with the allelic exchange vector pKSV7 (Smith and Youngman, 1992) that had been digested with the same restriction enzymes, resulting in plasmids pKSV7Δ*lmo0644*, pKSV7Δ*lmo0927*, pKSV7Δ*lmo2553*, pKSV7Δ*lmo2554* and pKSV7Δ*lmo2555*. These plasmids were introduced by electroporation (Park and Stewart, 1990) into strain 10403S and a previously described allelic exchange method (Camilli *et al*., 1993) was used to create strains 10403SΔ*lmo0644*, 10403SΔ*lmo0927*, 10403SΔ*lmo2553*, 10403SΔ*lmo2554* and 10403SΔ*lmo2555*. Plasmids pKSV7Δ*lmo2553* and pKSV7Δ*lmo2555* and strain 10403SΔ*lmo0644* were used to create double mutants 10403SΔ*lmo0644/2553* and 10403SΔ*lmo0644/2555* respectively. Deletions were confirmed by PCR using primer pairs listed in Table 3, which bind outside the region of homology used for allelic exchange. For complementation analysis, genes *lmo0644*, *lmo0927* and *lmo2555* were cloned with their native promoter into the *L. monocytogenes* single-site integration vector pPL3 (Gründling *et al*., 2004), while genes *lmo2553* and *lmo2554* were cloned under the control of the hyper-spac promoter into the integration vector pHPL3 (Gründling *et al*., 2004). Briefly, primers 5-BamHI-LMO0644_pPL3 and 3-KpnI-LMO0644_pPL3 (for complementation of *lmo0644*), 5-SalI-LMO0927_pPL3 and 3-KpnI-LMO0927_pPL3 (for complementation of *lmo0927*), 5-BamHI-LMO2553_pPL3HSPAC and 3-KpnI-LMO2553_pPL3HSPAC (for complementation of *lmo2553*), 5-BamHI-LMO2554_pPL3HSPAC and 3-KpnI-LMO2554_pPL3HSPAC (for complementation of *lmo2554*) and 5-BamHI-LMO2555_pPL3 and 3-KpnI-LMO2555_pPL3 (for complementation of *lmo2555*) were used to amplify relevant fragments from chromosomal DNA of strain 10403S. The resulting PCR products were digested with the restriction enzymes KpnI and BamHI or KpnI and SalI for the *lmo0927* PCR product and ligated either with pPL3 or with pHPL3, which were digested with the same restriction enzymes, resulting in plasmids pPL3-*lmo0644*, pPL3-*lmo0927*, pHPL3-*lmo2553*, pHPL3-*lmo2554* and pPL3-*lmo2555*. Plasmids pPL3-*lmo0644*, pHPL3-*lmo2553*, pHPL3-*lmo2554* and pPL3-*lmo2555* were electroporated into the relevant deletion strains resulting in complementation strains listed in Table 2. Plasmid pPL3-*lmo0927* was introduced into strain 10403SΔ*lmo0927* by conjugation (Lauer *et al*., 2002) resulting in strain 10403SΔ*lmo0927* pPL3-*lmo0927*. As controls, empty pPL3 and pHPL3 vectors were introduced into wild-type 10403S and relevant deletion strains by electroporation or conjugation for strain 10403SΔ*lmo0927*. For expression of C-terminally His-tagged Lmo0644 and Lmo0927 proteins under their native promoter control, plasmids pPL3-*lmo0644His6* and pPL3-*lmo0927His6* were constructed. The C-terminal His6 tag was introduced by PCR using primer pair 5-BamHI-LMO644_pPL3/3-SalI-LMO0644-C-His and 5-PstI-LMO0927-withP/3-SalI-LMO0927-C-His and 10403S chromosomal DNA. The resulting PCR products were cut with BamHI and SalI (for *lmo0644His6*) or PstI and SalI (for *lmo0927His6*) and inserted into vector pPL3 cut with the same enzymes. As control, the *S. aureus ltaS* gene, annotated as *SAV0719* in the MU50 genome, was also cloned as C-terminal His6 fusion under its native promoter into the *L. monocytogenes* integration vector pPL3. Plasmid pPL3-*ltaS*_*SA*_*His6* was constructed by amplifying *ltaS* from *S. aureus* RN4220 chromosomal DNA using primer pair 5-BamHI + P SAV0719 and 3-KpnI-His6-719 and the PCR product was cut and cloned as BamHI and KpnI fragment into vector pCL55 (Lee *et al.*, 1991), resulting in plasmid pCL55-*ltaS*_*SA*_*His6*. The BamHI/KpnI fragment was subsequently excised and cloned into pPL3, resulting in plasmid pPL3-*ltaS*_*SA*_*His6*. Plasmids for expression of His-tag protein fusions were initially recovered in *E. coli* strain XL1-Blue and subsequently introduced by electroporation into the *L. monocytogenes* strain 10403S, resulting in strains 10403S pPL3-*lmo0644His6* and 10403S pPL3-*lmo0927His6*, 10403S pPL3-*ltaS*_*SA*_*His6*. The DNA sequences of all inserts were verified by automated fluorescence sequencing at the MRC Clinical Sciences Centre Genomics Core Laboratory, Imperial College London.

**Table 3:** Primers used in this study.

Number	Name	Sequence
ANG383	5-KpnI-LMO0644	GG**GGTACC**GGAGGAAACGGCATCAAAACCTAAATAAGCAAAG
ANG384	5-int-LMO0644	CGTAATGGTAAATTAATAATTAGTAAAAAATAAAATAAAATCAA
ANG637	3-int-LMO0644–10403S	TTACTAATTATTAATTTACCATTACGAGACGAAGATAAATAA
ANG386	3-BamHI-LMO0644	CG**GGATCC**GGCGCACTGTTTATCGTTATCGTTGGCTAC
ANG376	5-KpnI-LMO0927	GG**GGTACC**GTAGCTCTTCTTATGAAGCAAAGAAAATCAGTG
ANG377	5-int-LMO0927	AGTTGATTTTTTCGTTTGGATTTTTATTTTCCAATCCTTCAT
ANG378	3-int-LMO0927	AAAATCCAAACGAAAAAATCAACTGATTCATCCGATAAATAA
ANG379	3-BamHI-LMO0927	CG**GGATCC**CGTTATCGTGCCACAAGTGTTATTTTGTGG
ANG544	5-KpnI-LMO2553	GG**GGTACC**GTTTTGAGAAATCGGATATCACGCATTACC
ANG545	5-int-LMO2553	AGGTGTTTTTGCAAATAAGTTTTTCTTTGCGCCTCCACTCAT
ANG546	3-int-LMO2553	AAAAACTTATTTGCAAAAACACCTGCAAAAAATTTACCATAG
ANG547	3-BamHI-LMO2553	CG**GGATCC**CGCAATGATTCCCTCTAAGTGAGTTGGGAT
ANG551	5-KpnI-LMO2554	GG**GGTACC**TTACTTGCATTATATTGCGAAAGGTAAAATTTTG
ANG552	5-int-LMO2554	TCGATCCTCTGATGCCGAAGATAGCATTGTCAACTTAATCAC
ANG553	3-int-LMO2554	CTATCTTCGGCATCAGAGGATCGACTAGCTGAAATATGGTT
ANG554	3-BamHI-LMO2554	CG**GGATCC**AGTGTACTCAGCTCCACCGGCCCCGCCTGG
ANG558	5-KpnI-LMO2555	GG**GGTACC**GCTTTGATTCTTGTAAAGCGGCTATCGATG
ANG559	5-int-LMO2555	AACGTGTGTAGAGTAGGTATCCGTAAAAATCCCTATATTCAT
ANG560	3-int-LMO2555	ACGGATACCTACTCTACACACGTTCAAAGGAAAGAGAGGTCA
ANG561	3-BamHI-LMO2555	CG**GGATCC**CCCTAATAAATCCAGGGTTATCTACTTCTTTCAC
ANG651	5-BamHI-LMO0644_pPL3	CG**GGATCC**TTTGTCTCCTACCTTTTTACATTCTTC
ANG652	3-KpnI-LMO0644_pPL3	GG**GGTACC**TTATTTATCTTCGTCTCGTAATGGTAAATTG
ANG653	5-SalI-LMO0927_pPL3	ACGC**GTCGAC**CTAGCAGACTTCCATTCCAAATGGTTC
ANG654	3-KpnI-LMO0927_pPL3	GG**GGTACC**TTATTTATCGGATGAATCAGTTGATTTTTTC
ANG659	5-BamHI-LMO2553_pPL3HSPAC	CG**GGATCC**CAAGGATTATTAACGAAGGAGTGAAAG
ANG660	3-KpnI-LMO2553_pPL3HSPAC	GG**GGTACC**CTATGGTAAATTTTTTGCAGGTGTTTTTGC
ANG661	5-BamHI-LMO2554_pPL3HSPAC	CG**GGATCC**ACGTTCAAAGGAAAGAGAGGTCATC
ANG662	3-KpnI-LMO2554_pPL3HSPAC	GG**GGTACC**TCACTCCTTCGTTAATAATCCTTGAT
ANG663	5-BamHI-LMO2555_pPL3	CG**GGATCC**GGTTCTAACGGTAAAGCGTAAGACGAAC
ANG664	3-KpnI-LMO2555_pPL3	GG**GGTACC**TTAATCACGCCGCGATGACCTCTCTTTCC
ANG673	3-SalI-LMO0644-C-His	ACGC**GTCGAC**TTAGTGATGGTGATGGTGATGACCTTTATCTT CGTCTCGTAATGGTAAATTG
ANG674	5-PstI-LMO0927-withP	AA**CTGCAG**CTAGCAGACTTCCATTCCAAATGGTTC
ANG676	3-SalI-LMO0927-C-His	ACGC**GTCGAC**TTAGTGATGGTGATGGTGATGACCTTTATCGGAT GAATCAGTTGATTTTTTC
ANG086	5-BamHI + P SAV0719	CG**GGATCC**GGAATAGAATATAGAATGCAATTAGAAATG
ANG419	3-KpnI-His6-719	GG**GGTACC**TTAGTGATGGTGATGGTGATGACCTTTTTTAGAG TTTGCTTTAGGTCCTG
Primers for verifying deletion strains		
ANG380	5-check-LMO0927	CTTTAACATATGATTCCTCCTTGTAAC
ANG381	3-check-LMO0927	CTTTCTACTTTTGCAAATAATGAATTTCAAATC
ANG387	5-check-LMO0644	CGGCATCGTCCGTTGCGGATCTTTCAC
ANG388	3-check-LMO0644	GCCGCGCCGCACTGGAAGATACGATGAC
ANG548	5-check-LMO2553	GTAAAAGGTCAGGGTGTGGCATCAG
ANG549	3-check-LMO2553	CAACTTTTTTTATATTCTCTACTTCACC
ANG555	5-check-LMO2554	TAGGTCTTTTAGGTAAGCGAATTG
ANG556	3-check-LMO2554	CTCCTGCACCAAAAACGATACAAC
ANG562	5-check-LMO2555	ACTGAAGGACTTGTAGAAGACCTG
ANG563	3-check-LMO2555	CTAGTCGATCCTCTGAATAATAAG

Restriction sites in primer sequences are underlined and shown in bold.

### LTA and protein detection by Western blot

Lipoteichoic acid and protein detection by Western blot was undertaken essentially as previously described (Gründling and Schneewind, 2007b). In brief, for sodium dodecyl sulphate (SDS)-polyacrylamide gel electrophoresis (PAGE) and Western blot analysis of cell-associated LTA and His-tagged proteins, 1 ml of overnight culture was mixed with 0.5 ml of 0.1 mm glass beads and lysed by vortexing for 45 min in the cold. Glass beads were sedimented by centrifugation at 200 *g* for 1 min, and 0.5 ml of the resultant supernatant transferred to a fresh tube. Bacterial debris and LTA were sedimented by centrifugation at 17 000 *g* for 15 min and suspended in protein sample buffer containing 2% SDS normalized for OD_600_; that is, samples from a culture with an OD_600_ of 2 were suspended in 50 μl of sample buffer. Samples were boiled for 20 min, centrifuged at 17 000 *g* for 5 min and 10 μl of samples loaded onto SDS-PAA gels. To determine the amount of LTA shed into the culture medium, 500 μl of culture was first centrifuged at 17 000 *g* for 5 min to pellet bacteria. Culture supernatant (100 μl) was removed, mixed with 100 μl of 2× protein sample buffer, boiled for 30 min and insoluble material removed by centrifugation at 17 000 *g* for 5 min. Supernatant samples were normalized based on OD_600_ of 2, in that 10 μl of a culture of OD_600_ of 2 was loaded. To determine if the His-tagged proteins were shed into the supernatant, 1.4 ml of culture was centrifuged at 17 000 *g* for 10 min to pellet the bacteria. One millilitre of the supernatant was transferred to a new tube, mixed with 100 μl of 100% trichloroacetic acid (TCA), vortexed, incubated on ice for 1 h and centrifuged for 10 min at 17 000 *g*. The supernatant was aspirated and the TCA precipitated pellet was washed twice with 1 ml of ice-cold acetone. Between wash steps, samples were incubated on ice for 1 h and debris collected by centrifugation as described above. After the final centrifugation step, pellets were air dried and suspended in 2× protein sample buffer normalized for OD_600_; that is, samples from a culture with an OD_600_ of 2 were suspended in 100 μl of sample buffer. The samples were boiled for 30 min and 10 μl analysed by Western blot. LTA samples were routinely loaded onto 15% SDS-PAA gels and probed with polyglycerolphosphate-specific LTA antibody (Clone 55 from Hycult biotechnology) and HRP-conjugated anti-mouse IgG (Cell Signalling Technologies, USA) used at 1:2000 and 1:10 000 dilutions respectively. His-tagged protein samples were routinely loaded onto 10% SDS-PAA gels and probed with HRP-conjugated His-tag-specific antibody (Sigma) used at a 1:10 000 dilution and Western blots were developed by enhanced chemiluminesce (ECL). Western blots were performed with at least three independently grown cultures in at least two independent experiments and representative images are shown.

### Growth curves and determination of colony-forming units

Wild-type and mutant *L. monocytogenes* cultures were grown overnight at 30°C in 4 ml of BHI medium. Next day, cultures were diluted to a starting OD_600_ of 0.07 into 25 ml of BHI medium, incubated with shaking at 30°C or 37°C and OD_600_ values determined at timed intervals. Growth curves were performed in duplicate and representative graphs are shown. To determine the number of colony-forming units (cfu) per ml of culture normalized for OD_600_ of 2, the optical density of overnight cultures grown at 30°C was determined, cultures normalized based on OD_600_ readings and a dilution series prepared in phosphate-buffered saline (PBS) pH 7.4. Fifty microlitres of appropriate dilutions were plated in duplicate onto BHI plates and plates incubated at 30°C or 37°C. Colonies were enumerated after 24 or 48 h growth for wild-type and 10403SΔ*lmo0927 L. monocytogenes* strains, respectively, and average values plus standard deviations for three independently grown cultures are given.

### Wide-field and transmission electron microscopy

For wide-field microscopy, strains 10403S and *10403S*Δ*lmo927* were grown overnight at 30°C in 4 ml of BHI medium. Next day, cultures were diluted 1:100 or 1:50 (for strain 10403SΔ*lmo0927* for subsequent growth at 37°C) into 25 ml of fresh medium and grown either at 30°C or 37°C. Eight and a half hours after back dilution, culture aliquots were removed, washed once with 1 ml of PBS pH 7.4 buffer and viewed under a 100× objective on a Nikon Elipse E600 microscope and images taken with a Nikon DXM1200 digital camera. Experiments were performed with at least three independently grown cultures in at least two independent experiments and representative images are shown.

Samples for TEM were prepared similar as described in Thomaides *et al*. (2001). Briefly, *L. monocytogenes* strains were grown overnight at 30°C, back-diluted between 1:100 and 1:25 into 200 ml of BHI medium and grown for 3.5 or 8.5 h at 30°C or 37°C as indicated in the text. Bacteria from an equivalent of a 100 ml culture with an OD_600_ of 0.5 were collected by centrifugation for 10 min at 8000 *g*, washed twice with 10 ml of 0.2 M sodium cacodylate buffer pH 7.1 and finally suspended in 2 ml of 0.2 M sodium cacodylate buffer pH 7.1 containing 4% glutaraldehyde. Bacteria were fixed for 4 h at 4°C without shaking and subsequently collected by centrifugation for 5 min at 10 000 *g*. Bacteria were suspended in 1 ml of 0.2 M sodium cacodylate buffer pH 7.1, transferred to 35 mm round tissue culture dishes, overlaid with 1 ml of 0.2 M sodium cacodylate buffer pH 7.1 containing 4% glutaraldehyde and incubated for 1 h at room temperature. After this second fixation step, bacterial layers were washed six times with 2 ml of 0.2 M sodium cacodylate buffer pH 7.1 and processed for electron microscopy as previously described (Thomaides *et al*., 2001). Images were taken on an FEI Tecnai GZ transmission electron microscope at the Henry Wellcome Trust Imaging Centre, St Mary's Campus, Imperial College London.

### Membrane lipid extraction and detection of glycolipids by TLC

For *L. monocytogenes* membrane lipid extraction and glycolipid analysis, bacteria from 200 ml of cultures grown for 20–24 h at 30°C were collected by centrifugation for 10 min at 8000 *g*. Bacteria were washed once with 10–20 ml of ice-cold 0.1 M sodium citrate buffer pH 4.7, suspended in 3 ml of 0.1 M sodium citrate buffer pH 4.7 and dispensed into three 2 ml Fast Prep tubes containing 0.1 mm glass beads (∼0.5 ml). Bacteria were lysed and lipids extracted as previously described using a modified Bligh-Dyer method (Kates, 1972; Gründling and Schneewind, 2007b). Dried lipids were suspend either in chloroform or in a 1:1 chloroform : methanol mix at a concentration of 50 mg ml^−1^. Ten microlitres corresponding to a total of 500 μg of lipids were spotted onto pre-run silica gel Å60 plates (Macherey-Nagel), lipids separated using a chloroform : methanol : H_2_O (65:25:4) solvent system and glycolipids visualized by spraying plates with 0.5% α-naphthol in 50% methanol and then with 95% H_2_SO_4_ (Gründling and Schneewind, 2007b; Kates, 1972). Experiments were performed with at least three independently grown cultures in at least two independent experiments and representative images are shown.

### Lipid analysis by MALDI mass spectrometry

For MALDI analysis to determine and confirm the structure of different glycolipids, a total of 4 mg of lipids were spotted (4 × 20 μl) and separated by TLC as described above and different lipids were further purified after scraping the silica gel from appropriate areas. Areas containing glycolipids were determined by developing one lane run in parallel with α-naphthol and H_2_SO_4_ and lipids were extracted from the silica gel with chloroform/methanol as previously described (Gründling and Schneewind, 2007b). Dried lipids were suspended in 10 μl of 0.5 M 2,5-dihydroxybenzoic acid (DHB) MALDI matrix dissolved in 1:1 methanol : chloroform and 1 μl was spotted directly onto MALDI plates or diluted 1:10 using 0.5 M DHB matrix and 1 μl spotted. Spotted MALDI plates were run on a MALDI micro MX™ machine (Waters, UK) available at the Proteomics Facility at Imperial College London. Using an automated program, 10 spectra were recorded for each spot in the reflector positive ion mode. As calibration standard, 25–50 pmoles of bradykinin peptide standard (Sigma) with an absolute mass of 757.3997 (M+H^+^) was spotted in α-cyano-4-hydroxycinnamic acid (CHCA) matrix, which was suspended at 10 mg ml^−1^ in 70% acetonitrile 0.1% TFA. Mass signals for lipids were manually corrected for observed mass difference of the internal peptide standard. Representative data from two independent experiments are shown.
