# Supplementary material for: Two-enzyme systems for glycolipid and polyglycerolphosphate lipoteichoic acid synthesis in Listeria monocytogenes
Source: Mol Microbiol. 2009 Aug 24;74(2):299–314. doi: 10.1111/j.1365-2958.2009.06829.x (PMC2764115; doi:10.1111/j.1365-2958.2009.06829.x)
Supplement: Supplementary file 1 [file mmi0074-0299-SD1.pdf]

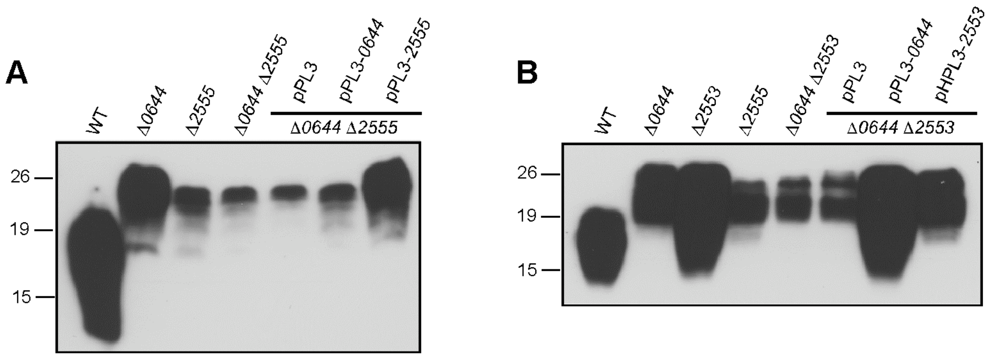

Supplemental Fig. 1. Western-blot detection of cell wall associated LTA in *Listeria monocytogenes* wild-type, deletion and complementation strains: (A) 10403S (WT), 10403SΔ*lmo0644* (Δ*0644*), 10403SΔ*lmo2555* (Δ*2555*), 10403SΔ*lmo0644* Δ*lmo2555* (Δ*0644* Δ*2555*), 10403SΔ*lmo0644* Δ*lmo2555* pPL3 (pPL3), 10403SΔ*lmo0644* Δ*lmo2555* pPL3-*lmo0644* (pPL3-*0644*), and 10403SΔ*lmo0644* Δ*lmo2555* pPL3-*lmo2555* (pPL3-*2555*); (B) 10403S (WT), 10403SΔ*lmo0644* (Δ*0644*), 10403SΔ*lmo2553* (Δ*2553*), 10403SΔ*lmo2555* (Δ*2555*), 10403SΔ*lmo0644* Δ*lmo2553* (Δ*0644* Δ*2553*), 10403SΔ*lmo0644* Δ*lmo2553* pPL3 (pPL3), 10403SΔ*lmo0644* Δ*lmo2553* pPL3-*lmo0644* (pPL3-*0644*), and 10403SΔ*lmo0644* Δ*lmo2553* pHPL3-*lmo2553* (pHPL3-*2553*). Positions of protein standards (in kDa) are shown on the left.
